# Medical and Physical Intelligence

**Published:** 1820-05

**Authors:** 


### Medical and Physical Intelligence.

TRANSACTIONS OF SCIENTIFIC SOCIETIES.

*ROYAL SOCIETY of Edinburgh, January* 17.—Dr. FERGUSSON, Inspector of Hospitals, read a paper *On the Nature and Properties of the Marsh-Poison,* as known under the titles of *Marsh Miasmata* and *Malaria.*

The author endeavoured to prove, from a reference to the medical topography of various places in the south of Europe and the West Indies, that the universally-received opinions of aqueous and vegetable putrefaction, single or combined, being the sources of this poison, were unfounded; that putrefaction, under any shape, had no effect in producing it; that it never emanates from water in bulk, however putrid that water may be, nor is necessarily an exhalation from marshes: but some modification of the state of the atmosphere by heat and moisture, being the product of a highly-advanced stage of the drying process in absorbent soils that had previously and recently been saturated with water. The illustrations were principally taken from the countries where the author had served with the British armies during the last twenty-five years, and exhibited a great variety of observations in support of the opinions the paper professed to advocate. Other properties of the marsh-poison, such as its particular adherence to, and attraction for, lofty umbrageous trees and rising grounds in the neighbourhood of swamps; its concentration in ravines, hollows, or leeward localities; its absorption from passing over water, and rarefaction or dissipation by the sun's heat, and regular currents of wind, were also pointed out and illustrated. In the course of the paper, the author, while treating of the effects of the marsh-poison, was led to consider its extreme and most baleful product,—the yellow-fever of the tropics; and he endeavoured to show the non-contagious nature of that disease, by a series of forcible arguments. It concluded with some observations on the mode of reception of the marsh-poison into the human constitution, whether by the lungs, the stomach, or the skin, which last the author seems to think the most probable channel; and he supported his opinion by some illustrations taken from the plague of the Levant,—the peculiar idiosyncracy of the African or Creole negroes.

---

*Royal Academy of Sciences of Paris.*—In a memoir lately read to this learned body, by M. Dutrochet, *On the Structure and Regeneration of Feathers*, the author introduced some observations on the structure of the skin of vertebral animals in general, that seem to throw considerable light on many morbid states of this organ frequently witnessed in man.

The skin of vertebral animals, according to M. Dutrochet, presents, proceeding from the exterior to the interior, the following layers:

1. The epidermis.
2. The corneous teguments of the papillæ.
3. The layer of colouring matter.

The two latter layers, sometimes separate, are often confounded, and frequently in a state of softness, which prevents their being distinguished from each other, form what has been called the *mucous body*.

4. The epidermic membrane of the papillæ.

This membrane, absolutely imperceptible in the greater proportion of circumstances, may be very easily seen on the bulb of the feathers and under the scales of the limbs of birds: it is even seen below the scales of fish. It resembles in every respect the exterior epidermis.

5. The papillary layer.
6. The dermis.

The corneous envelopment ordinarily receives its colour from the layer of colouring matter with which it is in contact; but it, under some circumstances, remains colourless. Thus, the nails, which are ordinarily of the hue of the layer of colouring matter in brute animals, are colourless in the negro. The corneous substance of the hair is equally colourless in them as in whites. These facts may be added to some others which serve to prove that the corneous substance is perfectly distinct from the colouring matter, although it is often mingled with it. This corneous layer gives origin to hair, feathers, and scales, as well as horns. It does not exist in a continuous layer, but as an assemblage of small teguments; and is present in all vertebral animals. Hair and nails are not the only productions which attest the existence of this corneous envelopment in the skin of man. There are accidental productions, which prove that it exists even in regions of the skin where it does not manifest itself in a sensible manner in general. Such are horns, and similar productions, so often seen on the surface of the skin of man.

The epidermic membrane of the papillæ is not ordinarily perceptible in man. It nevertheless exists under the nails, and it becomes thickened when these organs are separated from the papillary layer they ordinarily cover. There is one case in which this epidermic membrane manifests itself in man: it is in that of *tatouing*. In this operation, a colouring substance is introduced below the epidermis by a multitude of minute punctures, and it remains there without alteration throughout the remainder of life. Now, this foreign matter, although subjacent to the epidermis, is certainly not in immediate contact with the papillary layer, on which it would occasion morbid effects by its qualities as a foreign body. It is certain, that this colouring matter is contained in the interval which separates the exterior epidermis from the epidermic membrane of the papillæ, and that it is mingled with the mucous body.

From a memoir read to the same body by M. Chateauneuf, *On the Diseases of the Lungs which have been observed at Paris*, in 1816, 1817, and 1818, it would appear, that pulmonary phthisis is not so prevalent in that city as it was formerly. In the three years just mentioned, the total number of deaths amounted to 62,441: of which, 604 were caused by asthma; 1,894, by pleurisies and peripneumonies; 4,459, by bronchial catarrhs; and 6,971, by pulmonary phthisis: total number from diseases of the lungs, 13,928.

Diseases of the lungs cause, then, about two-ninths of the deaths at Paris; and they divide amongst them their victims in the following manner:

| | |
|---|---|
| Asthma carries off nearly | 1 in 100 |
| Inflammations of the respiratory organs | 1 — 33 |
| Pulmonary catarrhs | 1 — 15 |
| Pulmonary phthisis | 1 — 9 |

Bayle had calculated that, a few years since, pulmonary phthisis destroyed one in five of patients in general.

Our Bills of Mortality are not drawn up with sufficient exactness to permit us to calculate on them with much confidence, but it appears from them, that the number of deaths in London in 1817, 1818, and 1819, amounted to 58,901; of whom, 12,281 are stated to die from *consumption*, which is very nearly as great, in proportion to the total number of deaths, as that from diseases of the lungs altogether in Paris; and it corresponds very nearly with the calculation made by Sydenham. But it must be remarked, that, with the exception of a small proportion of cases of *asthma* (which probably include most cases of chronic disease of the heart), and of *dropsy in the chest*, there are no other cases in the Bills of the last year of deaths which can be referred to diseases of the lungs; so that it is probable that *consumption* includes all the deaths arising from disease of those organs, excepting those termed asthma and dropsy: and this explanation makes the ratio of deaths from the diseases of the lungs to the total number, nearly equal in London and Paris. The above remarks apply also, with nearly the same precision, to the Bills of the two former years.

In those for 1818, we only find, besides those above mentioned, a very few from *palpitation of the heart* (only 7), and 15 from pleurisy; and in those for 1817, 4 from palpitation of the heart, and 22 from pleurisy. There is a moderate proportion under the head of *cough and hooping-cough*, also, in 1817 and 1818; but neither peripneumony nor catarrh are mentioned. There is always a high number, from 1000 to 1200, stated as *inflammation*, but of what organs we are ignorant: it most likely relates to inflammation of the external parts of the body.

It seems, then, from these considerations, fair to conclude, that *consumption*, in our Bills of Mortality, includes by far the greater proportion of the diseases of the lungs; and, consequently, that pulmonary phthisis is not of so frequent occurrence as might appear at a first view of the records under consideration.

As long as these Bills continue to be constructed under the direction of the *parish-clerks* and *searchers*, and regulated by the wills of the friends of the deceased, so long shall we be without any statistical records to which the medical philosopher can refer for any thing like correct information, in his researches respecting the influence of climate and civilization on the health of the inhabitants of our metropolis.

---

*The Preparation of the Laurel-water in general use in Italy.*—Take a pound of the fresh leaves of the *lauro-cerasus*, gathered in the beginning of the summer, the period at which they are the most succulent, and when the juice is most active; cut them in pieces, and put them in a glass retort with six ounces of water. Distil in a water-bath, and draw off three ounces. To be kept in a glass vessel, well closed. *Ordinary dose*, from four to six drops, diluted in two ounces of distilled water.

---

*Case of Premature Puberty.*—We extract the following case from the *Nouveau Journal de Medicine*, tome vii. as one of the most remarkable on record; for, in the greater number of instances, persons of this kind have either died at an early age, or else have not continued to fulfil the functions in general with ordinary vigour.

Marie-Augustine-Michel, a native of Pouné, department of the Seine et Oise, had the menses appear at the age of thirty months, and they have continued to recur regularly up to the present period, at which she is entering her fifty-third year, excepting during the times of pregnancy. At the age of eight years, she was four feet four inches in height, and her breasts fully developed; since which period she has not increased in height. She was married at the age of 27, and lost her first child after a difficult labour, from which she had a prolapsus of the uterus. She has since then aborted twice, and produced eight living children, two of which occurred as twins, in the last pregnancy but one. She is of a full habit of body, of a sanguineous temperament, and has constantly enjoyed perfect health; and now, in her fifty-third year, does not experience any symptom that indicates the approach of the cessation of menstruation. This case is related by Dr. Descuret.

### REPORT OF DISEASES.

HAD not the season of the latter part of the month, especially a very cold eastern wind, produced a recurrence of the maladies noticed in our last Report, we should have had to designate the period embraced by the present one as remarkably healthy. Although the first half of it was wet, and a little variable in respect to temperature, a rapid falling-off in the extent of disease was strikingly evident. But, as soon as the wind above mentioned appeared, a recurrence of inflammation of the mucous membranes of various organs was witnessed, appearing as catarrh, coryza, and in children especially, as diarrhœa. The larynx and trachea have been most frequently the express seat of the irritation when the respiratory organs have been affected. The mucous surfaces, properly speaking, on all occasions most readily sympathize with impressions on the skin; and we, by accident, have lately had occasion to witness in a very striking manner the effect of an eastern or north-eastern wind on the latter organ. A person who had borne the influence of fifty plates of a galvanic battery in full force when the wind blew from the south, without much sensible impression on thé skin, suffered acute pain and irritation of the same part from only fifteen plates of the same battery, under similar circumstances with respect to exercise, clothing, and diet; and this repeatedly. This, apparently, arose from a variation in the conducting power of the skin. But, it is not necessary to dwell on any further evidence of difference in the states of this organ during different winds; it has long been well known and attended to physicians.

The transference of irritation from fibrous structure to serous membranes, is much more rare than that just noticed; and, when it occurs, it is generally the pericardium that is the seat of the latter affection. This is a curious circumstance. One remarkable instance of this has just occurred to our observation. A girl, fifteen years of age, delicately educated, was seized, in the morning, with very severe pain in the fascia of the left fore-arm, the tendon of biceps flexor cubiti was also apparently affected, and the arm was bent and could not be straightened. About two hours afterwards, the other arm became affected in a similar manner. She was suspected by her parents of evincing deceit. She was going about the house in the course of the day. In the evening the pain in the arms disappeared, but was soon followed by intense acute pain in the left side, and a combination of symptoms as clearly indicative of pericarditis as we ever witnessed in any instance. She was bled in the arms that night and twice on the following day: on the two last occasions the blood showed a thick buffy coat. The deep pain over the left eye, which accompanied the thoracic disease from its onset, was the last symptom that remained. The pain in the arms never returned. She is now convalescent.

Hooping-cough is still rather prevalent, but not to near so great an extent as it was a few months since.

### METEOROLOGICAL JOURNAL.

By Messrs. WILLIAM HARRIS and Co. 50, Holborn, London.

*From March* 20 *to April* 19, *inclusive.*

| Day of Month. | Moon. | Rain gauge | THERM. | | | BAROM. | | De Luc's HYGROM. Dry | | Damp | | WIND. | | ATMOSPHERIC VARIATION. | | |
|---|---|---|---|---|---|---|---|---|---|---|---|---|---|---|---|---|
| Feb. 20 | | | 42 | 48 | 36 | 30·26 | 30·24 | | | 9 | 8 | E | NNE | Cloud. | Fine | |
| 21 | ☽ | | 44 | 49 | 36 | 30·17 | 29·97 | | | 8 | 9 | WNW | W | Fine | | Rain |
| 22 | | | 42 | 49 | 39 | 29·80 | 29·70 | | | 9 | 8 | N | W | Fine | | Rain |
| 23 | | | 45 | 49 | 42 | 29·54 | 29 18 | | | 9 | 7 | WNW | W | | Cloud. | Fine |
| 24 | | | 47 | 48 | 36 | 29·15 | 29·01 | | | 7 | 6 | NW | NE | Cloud. | Sho'ry | Cloud. |
| 25 | | | 40 | 44 | 33 | 29·23 | 29·60 | | | 5 | 6 | NE | NNE | Fine | | |
| 26 | | ·02 | 35 | 43 | 47 | 29·90 | 29·71 | | | 3 | 18 | WNW | W | Fine | Rain | |
| 27 | | ·01 | 51 | 53 | 47 | 29·82 | 29·85 | | | 19 | 17 | SW | WSW | Cloud. | Sleet | |
| 28 | | | 50 | 54 | 50 | 29·97 | 30 05 | | | 17 | 16 | WSW | SW | Fine | | |
| 29 | ○ | | 54 | 58 | 48 | 30·11 | 30·05 | | | 15 | 15 | SW | SSW | Fine | | |
| 30 | | | 52 | 56 | 42 | 29·95 | 30 04 | | | 13 | 11 | W | N | Foggy | Fine | |
| 31 | | | 46 | 58 | 44 | 30·00 | 29·93 | | | 9 | 17 | N | NW | Foggy | Fine | |
| Apr. 1 | | | 48 | 57 | 49 | 30·03 | 30·02 | | | 18 | 16 | WSW | WNW | Fine | | |
| 2 | | | 53 | 59 | 49 | 30·08 | 30·14 | | | 14 | 12 | W | WNW | Fine | | |
| 3 | | | 55 | 60 | 44 | 30·18 | 30·15 | | | 10 | 10 | NNW | ESE | Fine | Cloud. | Fine |
| 4 | | | 52 | 63 | 46 | 30·00 | 29·87 | | | 9 | 4 | S | SE | Fine | | |
| 5 | | | 55 | 63 | 47 | 29·80 | 29·64 | 3 | 1 | | | E | SW | Fine | Cloud. | Fine |
| 6 | ☾ | ·17 | 50 | 53 | 47 | 29·42 | 29·42 | | | 6 | 7 | SW | WSW | Rain | Cloud. | Cloud. |
| 7 | | ·02 | 51 | 53 | 38 | 29·48 | 29·52 | | | 7 | 9 | W | SW | Fine | Sleet | Cloud. |
| 8 | | ·12 | 45 | 50 | 39 | 29·44 | 29·29 | | | 7 | 6 | SW | SE | Fine | Cloud. | Rain |
| 9 | | | 45 | 50 | 39 | 29·42 | 29·54 | | | 6 | 6 | NNW | NW | Cloud. | Cloud. | Fine |
| 10 | | ·26 | 46 | 49 | 39 | 29·53 | 29 38 | | | 7 | 11 | SW | SSW | Cloud. | Rain | Cloud. |
| 11 | | | 49 | 54 | 42 | 29·50 | 29·64 | | | 15 | 14 | S | SSW | Clond. | Fine | Cloud. |
| 12 | ● | | 51 | 53 | 49 | 29·76 | 29·88 | | | 14 | 14 | SSW | SSW | Cloud. | | |
| 13 | | ·35 | 50 | 51 | 47 | 29·90 | 29·82 | | | 14 | 15 | E | E | Cloud. | Rain | Sleet |
| 14 | | ·45 | 49 | 50 | 41 | 29·60 | 29·70 | | | 16 | 14 | NE | N | Cloud. | Rain | |
| 15 | | | 45 | 51 | 42 | 29·91 | 29·90 | | | 13 | 11 | N | N | Fine | | |
| 16 | | | 52 | 55 | 52 | 30·05 | 30·18 | | | 10 | 12 | W | NW | Fine | Fine | Cloud. |
| 17 | | | 54 | 62 | 49 | 30·20 | 30·17 | | | 14 | 13 | NW | NNE | Cloud. | Fine | |
| 18 | | | 58 | 62 | 49 | 30·17 | 30·14 | | | 15 | 14 | NNE | ESE | Fine | | |
| 19 | | | 57 | 62 | | 30·15 | | | | 12 | | NNE | | Cloud. | Cloud. | |

The quantity of rain fallen in March is 26-100ths.

